# Sleep Disorders across the Lifespan: A Different Perspective

**DOI:** 10.3390/ijerph17239025

**Published:** 2020-12-03

**Authors:** Sergio Garbarino

**Affiliations:** 1 Department of Neuroscience, Rehabilitation, Ophthalmology, Genetics and Maternal-Infantile Sciences, University of Genoa, 16126 Genoa, Italy; sgarbarino.neuro@gmail.com; 2 Post-Graduate School of Occupational Medicine, Università Cattolica del Sacro Cuore, 00168 Rome, Italy

## 1. Introduction

Sleep constitutes a fundamental human behavior that results from the reorganization of brain functions. Humans spend almost one-third of their lives asleep. Sleep–wake activity is governed by a complex array of neural processes that are influenced by the environment and tightly integrated with other key biological processes, such as thermoregulation, hormone release, and feeding behaviors [1]. Obtaining a regular quantity and quality of sleep has been shown to benefit human physiology, wellbeing, and health through a number of different functions, such as the consolidation of memories [2], and the removal of free radicals [3] and neurotoxic waste [4]. Changes in sleep impact a wide variety of physiological systems, including those important for modulating weight, metabolism, immune function, and inflammation, which, in turn, influence the propensity for age-dependent diseases [5]. Moderate to strong relations exist between sleep quality and cognitive, physical, and mental health, and these relations largely remain stable across the lifespan.

## 2. Sleep across the Lifespan 

In early life, sleep is a polyphasic dynamic process that changes every few weeks, becoming monophasic as children reach primary school age. The timing of sleep for primary school children is often associated with early bedtimes and early wake times. With the onset of puberty, the timing of sleep is delayed, leading to the characteristic ‘owl’-like behavior of adolescents. When an individual reaches their 20s, the reverse change occurs, with a return to earlier timing [6]. 

Changes in sleep duration and timing are accompanied by changes in sleep structure: the cyclic occurrence of periods of non-rapid-eye-movement (NREM) sleep and rapid-eye-movement (REM) sleep characterizes the organization of human sleep. In early infancy, sleep is predominated by REM, accounting for approximately 50% of sleep [7]; by the time children reach primary school age, REM sleep is reduced to around 20% of total sleep time, and there is an increase in NREM, primarily slow-wave sleep (SWS). With puberty, SWS and slow-wave activity (SWA) start to decline in parallel with synaptic density in cortical layers. This decline in SWS/SWA continues in the middle years of life, and these decreases vary in magnitude depending on gender [8]. A continued decline is seen in older adults, but at a slower rate [9,10]

In conjunction with the development and transformation of the organization of human sleep, circadian rhythms also change with age throughout the lifespan. Circadian rhythms emerge during early infancy [11]. The alignment of homeostatic and circadian rhythms changes physiologically in an age- and gender-related manner [12]: females are more likely to report disturbed sleep onset, but males more often report night-time awakenings [13]. 

## 3. The Relationship between Sleep and Health: Sleep Disorders and Chronic Diseases 

Some age-related decreases in health outcomes may be due to poorer sleep quality. “Sleep quality” is a multidimensional concept with complex effects and consequences across the lifespan. An accurate understanding of sleep quality is essential to understanding and supporting healthy aging across the lifespan [1]. Good sleep is not only essential for physical health and cognitive performance but also plays a critical role in emotional functioning [14].

Circadian dysfunction is observed in several brain disorders that emerge at different stages of life. Disruption of circadian rhythms is associated with a higher risk of brain disorders. For example, chronic shift-workers (workers with rotating schedules or consistent night shifts) are vulnerable to numerous diseases [15], including psychiatric disorders such as depression [16].

Sleep disturbances and circadian disruptions are associated with several neurodevelopmental disorders, including attention-deficit hyperactivity disorder (ADHD), autism spectrum disorders (ASDs), Prader–Willi syndrome (PWS), and Smith–Magenis syndrome (SMS).

Reductions in sleep quality, delays in the circadian phase, and evening preference are consistently reported in children and adults with ADHD [17] and may be correlated with the severity of ADHD symptoms. In adults with ADHD, a loss of circadian gene expression rhythms in the oral mucosa accompanies delays in cortisol rhythm and reduced-amplitude melatonin rhythms [18]. Abnormal melatonin rhythms are also reported in children with ADHD [17].

Sleep problems and dysfunction in circadian rhythms are very common in children with ASD [19], a neurodevelopmental disorder characterized by impairments in social communication, restricted interests, and repetitive behaviors. In this regard, the most consistent finding in prepubertal children, pubertal adolescents, and young adults with ASD is a reduced melatonin level in the evening [20].

Circadian disruptions (such as social jet-lag) during adolescence increase an individual’s vulnerability to substance use, and conversely, chronic exposure to these substances can lead to long-lasting changes in the circadian and sleep networks [21]. Moreover, adolescents with shorter sleep durations are more likely to use substances, including caffeine, nicotine, alcohol, and marijuana, and to engage in other risky behaviors [22,23].

During adolescence, many developmental changes occur in the frontostriatal reward circuitry, which includes the ventral striatum, dorsal striatum, and medial prefrontal cortex [24], with increases in synaptic pruning and myelination [25] as well as in the availability of dopamine in the limbic circuitry [26]. The combination of heightened activity in reward-related circuits and underdeveloped cognitive control centers contributes to greater emotionality, impulsivity, and reward-seeking behavior [27]. Adolescence is a time during which serious psychiatric disorders, including major depression, bipolar disorder, and schizophrenia, tend to emerge. A major component of many mood, anxiety, and psychotic disorders is a disrupted sleep–wake cycle [28].

Many of these selective synaptic pruning and refinement processes occur during sleep, and sleep is crucial for proper circuit maturation and long-term memory formation in humans and animals [29]. Excessive or insufficient pruning during adolescence may be linked to the development of psychiatric disorders [30].

In most people, there is a gradual shift toward an earlier chronotype from adolescence to adulthood. Between 20 and 50 years of age, men continue to have a greater evening preference than women on average [6], and these sex differences disappear after the age of 50 years, coinciding with menopause in women [6].

In females, menopause is a crucial point for the occurrence of sleep impairments, such as poor sleep quality and sleep deprivation, as well as sleep disorders such as OSA, restless legs syndrome, and insomnia [31], while in men, the sleep quality worsens steadily. Different concurrent decreases in SWS, SWA, and GH secretion already begin to emerge during the third decade of life [32]. 

Moreover, poor sleep in perimenopausal/postmenopausal women is associated with inflammation, cardiovascular and metabolic diseases [33], and mood disorders.

Our 24-h society depends on both shift-work and frequent travel across time zones. Shift-work has been associated with increased risks of cancer, obesity, heart disease, gastrointestinal dysfunction, sleep disorders, diabetes, and depression [34,35,36].

The traditional focus for hypertension prevention and management has been on reducing obesity and dietary sodium, increasing physical activity, prescribing antihypertensive medications, and treating sleep-disordered breathing [37]. However, epidemiological evidence [38] suggests that a sleep duration of less than 7 h contributes to hypertension development. A reverse J-shaped relationship exists between sleep duration and hypertension, and the strongest and most consistent associations are with habitual short sleep. Younger men and women present little risk, and receive some apparent benefit, from sleep durations that exceed 7 h. Furthermore, short sleep has been consistently more closely related to the risk for hypertension in women compared with men across the adult lifespan.

Older people (ages ~65 years and older) generally sleep less and have poorer sleep efficiency, increased night-time awakening, increased sleep latency, and greater levels of daytime sleepiness [39]. In older adults, less-robust circadian rhythms and more fragmented patterns of activity are risk factors for the development of dementia [40]. Moreover, the incidence of single-nucleotide polymorphisms (SNPs) in CLOCK and BMAL1 and in BMAL1 and PER1 are associated with increased risks of Alzheimer’s disease (AD) and Parkinson’s disease (PD), respectively [41,42]. Compared with healthy aged people, individuals with AD or PD have considerably lower melatonin rhythm amplitudes and excessive daytime sleepiness, as well as other sleep–wake cycle disturbances, such as later sleep onsets [43]. In individuals with PD, these sleep–wake symptoms often precede the development of motor or cognitive symptoms and may even be useful as a diagnostic biomarker [44], whereas, in patients with AD, sleep disruptions tend to begin after diagnosis [45].

## 4. Conclusions

Throughout the lifespan, sleep and circadian rhythm disruptions are strongly linked to the pathophysiology of specific psychiatric and neurodegenerative disorders, as well as cancer, cardiovascular disease, heart disease, hypertension and metabolic disease, obesity, gastrointestinal dysfunction, inflammation and infections, and sleep disorders. We need to better understand the bidirectional relationships between circadian rhythms, sleep, and environmental perturbations, such as stress [46] or abuse of drugs, and how circadian rhythms are intimately connected to neurotransmission, metabolism, immunity, and other processes for health promotion and disease prevention in everyday life and the workplace [47,48].
